# The Role of TGF-β1 and Mutant *SMAD4* on Epithelial-Mesenchymal Transition Features in Head and Neck Squamous Cell Carcinoma Cell Lines

**DOI:** 10.3390/cancers16183172

**Published:** 2024-09-16

**Authors:** Michael Bette, Laura Reinhardt, Uyanga Gansukh, Li Xiang-Tischhauser, Haifa Meskeh, Pietro Di Fazio, Malte Buchholz, Boris A. Stuck, Robert Mandic

**Affiliations:** 1 Institute of Anatomy and Cell Biology, Philipps-Universität Marburg, 35037 Marburg, Germany; 2 Department of Otorhinolaryngology, Head and Neck Surgery, University Hospital Marburg, Philipps-Universität Marburg, 35043 Marburg, Germany; 3 Department of Nuclear Medicine, Philipps-Universität Marburg, 35043 Marburg, Germany; 4 Clinic for Gastroenterology, Endocrinology and Metabolism, University Hospital, Philipps-Universität Marburg, 35043 Marburg, Germany

**Keywords:** epithelial–mesenchymal transition, TGF-β1, SMAD4, head and neck squamous cell carcinoma

## Abstract

**Simple Summary:**

Head and neck squamous cell carcinomas (HNSCCs) represent more than 90% of all malignancies in the upper aero-digestive tract. Their ability to metastasize is tightly associated with the patient’s survival. The process of epithelial–mesenchymal transition (EMT) is thought to be a central mechanism for invasion and metastasis. Here, we investigated the responsiveness of HNSCC cell lines and the HaCaT control cell line to the EMT master regulator TGF-β1 and observed major differences in the level of sensitivity to this cytokine. The more epithelial HNSCC cells and HaCaT control cells responded more extensively to TGF-β1 than the less epithelial HNSCC cells. Mutant SMAD4 was detected in the most mesenchymal HNSCC cell line and appears to contribute to mesenchymal features in HNSCC cells. These observations could help to explain the different histomorphological phenotypes of HNSCC tumors, which could be linked to the risk of metastatic spread.

**Abstract:**

The aim of the present study was to investigate possible differences in the sensitivity of HNSCC cells to known EMT regulators. Three HNSCC cell lines (UM-SCC-1, -3, -22B) and the HaCaT control keratinocyte cell line were exposed to transforming growth factor beta 1 (TGF-β1), a known EMT master regulator, and the cellular response was evaluated by real-time cell analysis (RTCA), Western blot, quantitative PCR, flow cytometry, immunocytochemistry, and the wound closure (scratch) assay. Targeted sequencing on 50 cancer-related genes was performed using the Cancer Hotspot Panel v2. Mutant, and wild type *SMAD4* cDNA was used to generate recombinant *SMAD4* constructs for expression in mammalian cell lines. The most extensive response to TGF-β1, such as cell growth and migration, β-actin expression, or E-cadherin (CDH1) downregulation, was seen in cells with a more epithelial phenotype. Lower response correlated with higher basal p-TGFβ RII (Tyr424) levels, pointing to a possible autocrine pre-activation of these cell lines. Targeted sequencing revealed a homozygous *SMAD4* mutation in the UM-SCC-22B cell line. Furthermore, PCR cloning of *SMAD4* cDNA from the same cell line revealed an additional *SMAD4* transcript with a 14 bp insertion mutation, which gives rise to a truncated SMAD4 protein. Overexpression of this mutant SMAD4 protein in the highly epithelial control cell line HaCaT resulted in upregulation of TGF-β1 and vimentin. Consistent with previous reports, the invasive and metastatic potential of HNSCC tumor cells appears associated with the level of autocrine secretion of EMT regulators such as TGF-β1, and it could be influenced by exogenous EMT cytokines such as those derived from immune cells of the tumor microenvironment. Furthermore, mutant SMAD4 appears to be a significant contributor to the mesenchymal transformation of HNSCC cells.

## 1. Introduction

Head and neck squamous cell carcinomas (HNSCCs), not including skin squamous cell carcinomas, represent the sixth most common cancer worldwide and account for more than ninety percent of all malignancies in the upper aero-digestive tract [1]. Approximately half of the HNSCC patients die from this disease [1]. Strongly associated with the patient’s prognosis is the propensity of HNSCC tumor cells to invade and metastasize [2]. Clinically, HNSCC tumors can exhibit a high or low invasive and metastatic potential. Similarly, HNSCC cells can show highly heterogeneous morphological phenotypes that appear related to a lower or higher tendency for invasion and metastatic spread [3]. The process of epithelial–mesenchymal transition (EMT) is essential for embryonic development as well as for physiological processes such as wound healing, but it also plays a major role in cancer progression [4,5,6,7,8]. A major dogma in cancer biology presumes that EMT is a prerequisite for the invasiveness and metastatic spread of tumor cells. From clinical observations, it is known that HNSCCs can be highly heterogeneous in terms of their ability to metastasize. The transforming growth factor-β (TGF-β) signaling pathway [9,10] plays a pivotal role in the process of EMT [5,11] and is associated with tumor progression and metastasis [12,13,14,15,16,17,18]. Similarly, SMAD4 (mothers against decapentaplegic homolog 4), a member of the TGF-β signaling pathway, acts as a common signaling mediator (co-SMAD) by binding two receptor (R)-SMADs followed by translocation to the nucleus and activation of gene transcription. Interestingly, *SMAD4* is frequently mutated in cancers, resulting in the promotion of invasion and metastasis [19,20]. Against this background, it appears obvious that the heterogenous cellular morphology of HNSCC cells, as seen in histopathological sections, could be induced by a more or less active TGF-β signaling pathway due to, e.g., TGF-β signaling associated cytokines derived from the tumor cell itself or from other cells in the tumor microenvironment (TME) [21]. Similarly, HNSCC cells in culture that are stripped from the TME also exhibit variable phenotypes, which could point to the presence or absence of autocrine secretion.

To further evaluate this aspect, we compared three HNSCC cell lines and the highly epithelial HaCaT keratinocyte cell line, regarding their epithelial and mesenchymal features and their response to the exogenously applied EMT master regulator TGF-β1 [22,23].

## 2. Materials and Methods

### 2.1. Cell Lines and Cell Culture

The spontaneously immortalized keratinocyte cell line HaCaT (RRID:CVCL_0038) [24] as well as the HNSCC cell lines UM-SCC-1 (RRID:CVCL_7707), UM-SCC-3 (RRID:CVCL_7740), and UM-SCC-22B (RRID:CVCL_7732) [25] were cultured in DMEM containing 10% fetal calf serum, supplemented with 2 mmol/L l-glutamine, 50 μg/mL gentamicin, 250 ng/mL amphotericin B, 100 U/mL penicillin, and 100 μg/mL streptomycin. Cells were incubated at 37 °C, 5% CO_2_, and 100% relative humidity. Cells were passaged using trypsin/EDTA solution (0.025% trypsin, 0.05% EDTA) after reaching 80–90% confluence.

### 2.2. Real-Time Cell Analysis

Real-time cell analysis (RTCA) was performed with the xCELLigence RTCA system (Agilent, Santa Clara, CA, USA). When in contact with the bottom of special gold-coated 96-well plates (E-Plate 96, Agilent, Santa Clara, CA, USA), adhesive cells interact with electrodes at the bottom of the well, inducing a measurable increase in impedance, which is measured every 15 min. Impedance was measured using the dimensionless parameter Cell Index (CI). The CI at any time is defined as (Rn-Rb)/(15Ω), where Rn is the impedance when cells are present in the well and Rb represents the impedance in the presence of the cell-free medium only. An increase in impedance depends on the interacting area size, cell number, adhesion, and cell size. Cells were grown as triplicates. Cytokines were added after reaching a cell index of at least 1. The E-plate was then further incubated for usually 4 to 5 days. To evaluate the typical growth pattern of each cell line, the cell lines UM-SCC-1 (5 × 10^3^/well), UM-SCC-3 (5 × 10^3^/well), UM-SCC-22B (20 × 10^3^/well), and HaCaT (5 × 10^3^/well) were seeded as triplicates in an E-plate, recording the cell index (CI) at 15 min intervals for at least 120 hr. TGF-β1 (recombinant human TGF-β1, cat# 240-B-002/CF, Bio-Techne, Minneapolis, MN, USA) was added to a final concentration of 10 ng/mL and the respective vehicle (4 mmol/L HCl) was used as a control. At the time of TGF-β1 addition, the cell index was normalized (normalized cell index (NCI)) and recorded for at least 96 hr. Data analysis was carried out using the RTCA software 1.2.1.1002 (ACEA Bioscience, San Diego, CA, USA).

### 2.3. Wound Closure (Scratch) Assay

To assess the migratory properties of the tested cell lines before and after exposure to TGF-β1, a wound closure assay, also known as the scratch assay, was performed. For this, cell lines were seeded in 6 well cell culture dishes (HaCaT, UM-SCC-1, and UM-SCC-3 at 200 × 10^3^ cells/well and UM-SCC-22B at 300 × 10^3^ cells/well) followed by incubation under standard conditions for 24 h. Subsequently, the medium was exchanged, 1.5 μL TGF-β1 (final concentration 10 ng/mL) or 1.5 μL vehicle control (4 mmol/L HCl) were added to the respective wells, and incubation was continued for 24 h. After reaching confluence, a 1000 μL pipette tip was used to produce a scratch in the cell layer. To assess migration into the cell-free area of the scratch (wound), the incubation was continued, and the marked area was photographed after 24 and 48 h. Image analysis was carried out with the ImageJ Software Version 1.54k [26]. The migration rate is usually judged by measuring the width of the “scratch”. However, due to effects that form during migration, the cell boundary can become very heterogeneous [27]. To account for this aspect, two values, the “percentage wound closure” and the “relative coastline length”, were calculated. Due to the loosening of the cell structure during migration, the “coastline length” increases [28].

### 2.4. Immunocytochemistry and Fluorescence Microscopy

Immunocytochemistry was deployed to investigate the influence of exogenously supplied TGF-β1 on the subcellular localization of E-cadherin (CDH1) and β-actin in the HaCaT, UM-SCC-1, UM-SCC-3, and UM-SCC-22B cell lines. Cells were grown on cover slips under standard culture conditions and treated with 10 ng/mL TGF-β1. After 72 h, cells were fixed for 5 min in cold methanol. Subsequently, cells were incubated in a buffer containing 3% BSA/0.3% NP40/PBS, followed by the addition of the primary antibodies. The following primary antibodies were deployed: E-cadherin (67A4) (1:50, mouse monoclonal, sc-21791, Santa Cruz Biotechnology, Inc. (SCBT), Dallas, TX, USA) and β-actin (1:500, mouse monoclonal, clone AC-74, A5316, Sigma). Donkey anti-mouse IgG-R was used as a secondary antibody (sc-2300, SCBT). DAPI (4′,6-diamidino-2-phenylindole) was deployed for nuclear counterstaining. Cover slips were positioned upside down onto a drop of fluorescent mounting medium (DakoCytomation, Carpinteria, CA, USA) on a microscope slide and sealed with nail polish. Microscopic images were documented by confocal microscopy (Leica TCS SP2, Leica Mikrosysteme Vertrieb GmbH, Wetzlar, Germany).

### 2.5. Flow Cytometry

The following primary antibody was used for the detection of E-cadherin during flow cytometry: Phycoerythrin (PE) mouse anti-human CD324 (E-cadherin) (1:20; cat#: 562870; BD Biosciences, San Jose, CA, USA). PE mouse IgG1, κ isotype (1:20; cat#: 555749; BD Biosciences) was used as the control antibody. Cells were incubated for 1 hr at room temperature on ice in the dark. Flow cytometry was performed with the BD FACSCalibur^TM^ flow cytometer (BD Biosciences, Franklin Lakes, NJ, United States). The flow cytometry results were analyzed using FlowJo™ v7.6.5 Software (BD Biosciences).

### 2.6. SDS-PAGE and Western Blot Analysis

Western blot analyses were performed as previously described [29] using whole cell lysates of HaCaT, UM-SCC-1, UM-SCC-3, and UM-SCC-22B cell lines. The following primary antibodies were used for protein detection typically at a dilution of 1:500: E-cadherin (67A4) (mouse monoclonal, sc-21791, SCBT), p-TGFβ RII (Tyr424) (rabbit polyclonal, sc-17007, SCBT), β tubulin (H-235) (rabbit polyclonal, sc-9104, SCBT), β-actin (mouse monoclonal, clone AC-74, A5316, Sigma). Secondary, horseradish peroxidase (HRP)-coupled antibodies were as follows: donkey anti-mouse IgG-HRP (1:2000; sc-2096; SCBT) and goat anti-rabbit IgG-HRP (1:2000; sc-2004; SCBT). Incubations with primary antibodies were carried out overnight at 4 °C, followed by incubation with the respective secondary antibody for 1 hr at room temperature. Immunoreactive bands were visualized by exposure to x-ray films (cat#: 5U8OM, Vision X Orthovision G, Agfa, Mortsel, Belgium) using the enhanced chemiluminescence method (Pierce™ ECL Western blotting-substrate, Thermo Fisher Scientific, Waltham, MA USA).

### 2.7. Quantitative Reverse Transcription Polymerase Chain Reaction

For mRNA quantification, RNA was extracted from cell lines using the RNeasy mini kit (Qiagen, Hilden, Germany). Following RNA extraction, cDNA was synthesized using the Transcriptor first strand cDNA synthesis kit (Roche Diagnostik GmbH, Mannheim, Germany), deploying the oligo(dT) primer. All steps were performed according to the manufacturer’s instructions. cDNA was used as a template for quantitative PCR analysis (QuantStudio^TM^ 5; Thermo Fisher Scientific GmbH, Dreieich, Germany), deploying the SYBR-Green master mix (Thermo Fisher Scientific GmbH). *RPLP0* (ribosomal protein lateral stalk subunit P0) and *HPRT1* (hypoxanthine-guanine phosphoribosyltransferase) were used as housekeeping genes. Primer design was carried out with Primer-BLAST [30]. The following primers (Invitrogen, Thermo Fisher Scientific GmbH) were used: *SMAD4*_2f1/1r2_total (5′-CTATGCCCGTCTCTGGAGGT-3′ and 5′-TCAATTCCAGGTGATACAACTCGT-3′), *CDH1*_f1/r1 (5′-TTGCACCGGTCGACAAAGGA-3′ and 5′-GAGTCCCAGGCGTAGACCAAG-3′), *TGFß1*_f1/r1 (5′-TATTGAGCACCTTGGGCACTGTT-3′ and 5′-CTCCCTTAACCTCTCTGGGCTTG-3′), *VIM*_f1/r1 (5′-CTTAGGGGCGCTCTTGTCCC-3′ and 5′-TTCAAGTCTCAGCGGGCTCC-3′), *ZEB1*_f1/r1 (5′-AGCCCTGCAGTCCAAGAACC-3′ and 5′-TCCGCATTTTCTTTTTGGGCGG-3′), *RPLP0*_f/r (5′-AGCCCAGAACACTGGTCTC-3′ and 5′-ACTCAGGATTTCAATGGTGCC-3′), *HPRT1*_f/r (5′-CCCTGGCGTCGTGATTAGTG-3′ and 5′-TCGAGCAAGACGTTCAGTCC-3′).

### 2.8. Cloning and Transfection of SMAD4 Constructs

For cloning purposes, specific *SMAD4* primers, which contain HindIII or EcoRI restriction sites, were designed via Primer-BLAST [30] (Smad4_f4: 5′-GGTGGTAAGCTTTCCAAAGGATCAAAATTGCTTCAGA-3′; Smad4_r4: (5′-GGTGGTGAATTCGGGCCCCAACGGTAAAAGAC-3′). RNA was isolated from HaCaT and UM-SCC-22B cell lines to obtain wild type and mutant *SMAD4* transcripts followed by cDNA synthesis and *SMAD4* DNA amplification using the REDTaq® ReadyMix^TM^ PCR Reaction Mix (Sigma Aldrich Inc., Saint Louis, MO, USA). PCR products were subjected to agarose gel purification (QIAquick gel-extraction kit; Qiagen). The pcDNA3.0 plasmid and the purified *SMAD4* amplicons were subjected to double digestion with HindIII/ EcoRI (NEB GmbH, Frankfurt am Main, Germany) followed by gel purification and ligation (T4-DNA Ligase; NEB GmbH, Frankfurt am Main, Germany) of *SMAD4* and pcDNA3 overnight at 16 °C. Competent DH5α bacterial cells (Invitrogen, Thermo Fisher Scientific) were transformed with the ligation products and grown on agar plates containing 100 µg/mL ampicillin (Carl Roth GmbH + Co, Karlsruhe, Germany) for selection. Bacterial clones were tested for the presence of an *SMAD4* insert by digestion after plasmid isolation or by direct PCR of bacterial clones. Plasmids were purified from bacterial cells (QIAprep spin miniprep kit and Qiagen plasmid Midi purification kit; Qiagen, Hilden, Germany) followed by sequence validation (4Base Lab, Reutlingen, Germany). Validated *SMAD4* plasmids together with the pcDNA3.0 plasmid as a control were transfected into HaCaT and UM-SCC-22B cell lines using Lipofectamine™ 2000 (Invitrogen, Thermo Fisher Scientific).

### 2.9. Targeted Sequencing

DNA of UM-SCC-1, UM-SCC-3, UM-SCC-22B, and HaCaT cell lines was extracted with the QIAamp DNA Mini kit (Qiagen), and DNA concentration was measured with the Qubit^TM^ 4.0 Fluorometer using the Qubit^TM^ dsDNA HS assay kit (MAN0017455, Thermo Fisher Scientific). Moreover, 10 ng DNA was used for the Ion AmpliSeq™ Cancer Hotspot Panel v2 (Thermo Fisher Scientific), and library preparation was performed with the Ion AmpliSeq™ library kit 2.0 (MAN0006735, Thermo Fisher Scientific). Sequencing was executed with the Ion Torrent Personal Genome Machine^TM^ (PGM^TM^, Thermo Fisher Scientific) according to the manufacturer’s protocols (MAN0014388, MAN0014579, MAN0017531, MAN0014583, Thermo Fisher Scientific) using the Ion 316^TM^ Chip Kit V2 (Thermo Fisher Scientific). Sequence analysis was performed with the Ion Torrent Suite^TM^ Software 5.8 and the Ion Reporter (Thermo Fisher Scientific).

### 2.10. Statistical Analysis

All tests were performed using the Graph PadPrism software (version 9.0.0 for MAC; GraphPad Software, Inc., San Diego, CA, USA). The following statistical analyses were used: The multiple unpaired *t*-tests followed by a false discovery rate (FDR) approach according to the two-stage step-up method of Benjamini, Krieger, and Yekutieli for real-time cell analysis (RTCA); the two-tailed unpaired *t*-test for flow cytometry analysis; the one-tailed, unpaired *t*-test for the scratch assay and RT-qPCR analysis. A Welch correction was always carried out if the variances of the two tested groups were significantly different. Data represent the mean ± standard deviation (SD), with *p* < 0.05 considered statistically significant. Statistical differences were indicated as *: *p* < 0.05, **: *p* < 0.01, ***: *p* < 0.001 and ****: *p* < 0.0001.

## 3. Results

Real-time cell analysis was performed to evaluate the growth characteristics of the three HNSCC cell lines UM-SCC-1, -3, -22B, as well as HaCaT control cells. All four cell lines exhibited different growth levels, with HaCaT cells growing only slowly in contrast to UM-SCC-1 cells that exhibited the fastest growth pattern (Supplementary Figure S1A). UM-SCC-3 cells were deployed to evaluate the range of responsiveness to different concentrations of TGF-β1, TGF-β2, and TGF-β3 (20 pg/mL, 0.2 ng/mL, 2 ng/mL, and 20 ng/mL) as well as hepatocyte growth factor (HGF) (100 pg/mL, 1 ng/mL, 10 ng/mL, and 100 ng/mL) (Supplementary Figure S1B). TGF-β1 in a range of 10–20 ng/mL was found to be effective for cell stimulation and was used in all following experiments.

**Figure 1:**
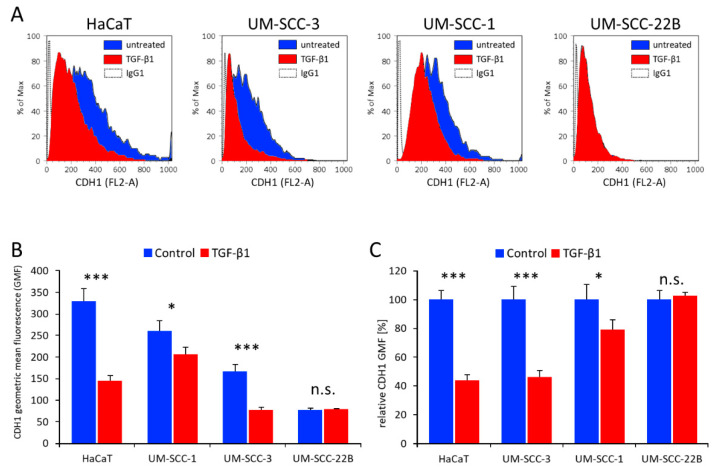
The effect of TGF-β1 on CDH1 surface expression in HNSCC and HaCaT cell lines. (**A**) Shown are representative flow cytometry histograms evaluating CDH1 surface expression (FL2-A channel for PE fluorescence) in cell lines treated with or without TGF-β1. (**B**) Comparison of absolute CDH1 geometric mean fluorescence (GMF) levels before and after TGF-β1 treatment. (**C**) Same data as in (B) depicting relative CDH1-GMF levels. Data represent the mean ± SD (*n* = 3), with *p* < 0.05 considered statistically significant. Statistical differences were indicated as *: *p* < 0.05, ***: *p* < 0.001, n.s.: not significant (see also Supplementary Figure S2).

Initially, the four tested cell lines were evaluated regarding the surface expression of the epithelial marker E-cadherin (CDH1) and its responsiveness to TGF-β1. Here, we could observe cell type-dependent CDH1 surface expression levels as well as differential responsiveness of CDH1 to TGF-β1. HaCaT cells exhibited the highest CDH1 surface levels consistent with a pronounced epithelial phenotype and were highly responsive to TGF-β1 whereas UM-SCC-22B cells showed lowest CDH1 surface levels with no measurable further loss after TGF-β1 treatment, consistent with a more mesenchymal phenotype. UM-SCC-3 and UM-SCC-1 cells exhibited an intermediate phenotype (Figure 1).

**Figure 2:**
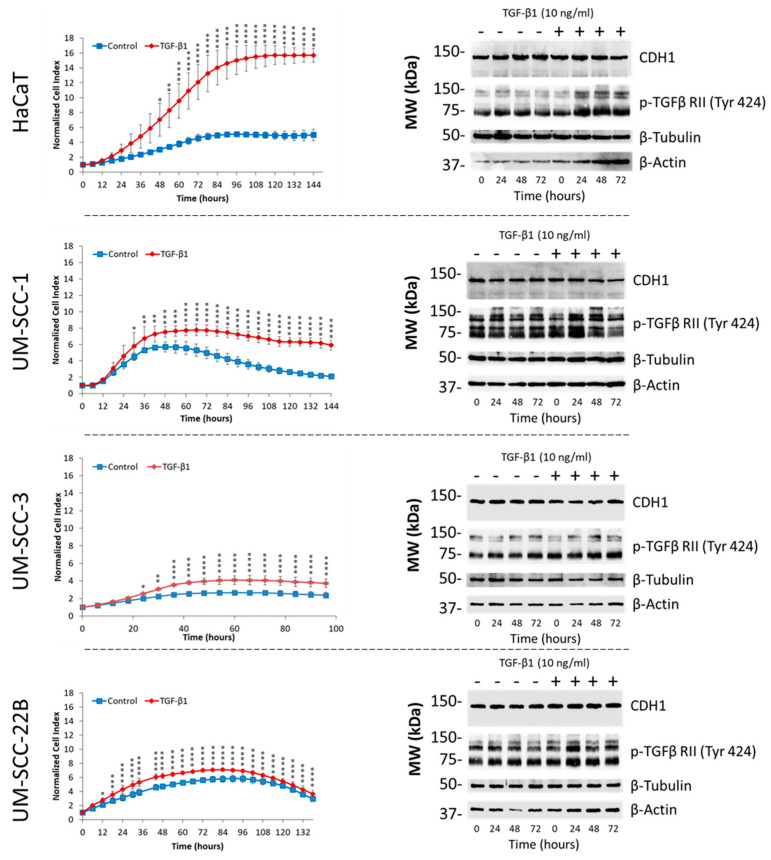
Differential responsiveness of HNSCC and HaCaT cell lines to exogenous TGF-β1. Real-time cell analysis (RTCA) demonstrates major differences in TGF-β1 mediated cell growth (normalized cell index) induction in the tested cell lines (left). Data represent the mean ± SD (*n* = 3), with *p* < 0.05 considered statistically significant. Statistical differences were indicated as *: *p* < 0.05, **: *p* < 0.01, ***: *p* < 0.001 and ****: *p* < 0.0001. Shown on the right is the protein expression of CDH1 (E-cadherin), pTGFβ RII (Tyr424), β-tubulin, and β-actin. Uncropped Western blots are shown in Figure S3.

After treatment with TGF-β1, HaCaT cells exhibited a dramatic rise in cell growth (normalized cell index) during RTCA analysis, whereas this response was less pronounced in the other three cell lines, particularly in UM-SCC-22B cells (Figure 2, left). Similarly, after TGF-β1 stimulation, a major rise in active TGF-β RII (p-TGFβ RII (Tyr424)) was noticed, particularly in HaCaT cells (Figure 2, right). However, the remaining cell lines, especially UM-SCC-1 and UM-SCC-22B, exhibited higher basal levels of active TGF-β RII, which did not appear to rise extensively after stimulation with exogenous TGF-β1 (Figure 2, right). Furthermore, a major β-actin induction was observed in HaCaT cells 48 and 72 hr after TGF-β1 exposure. Also, total cellular CDH1 appeared to be reduced 72 hr after the addition of TGF-β1 in HaCaT and UM-SCC-1 cells, which was less obvious in the other tested cell lines (Figure 2, right).

**Figure 3:**
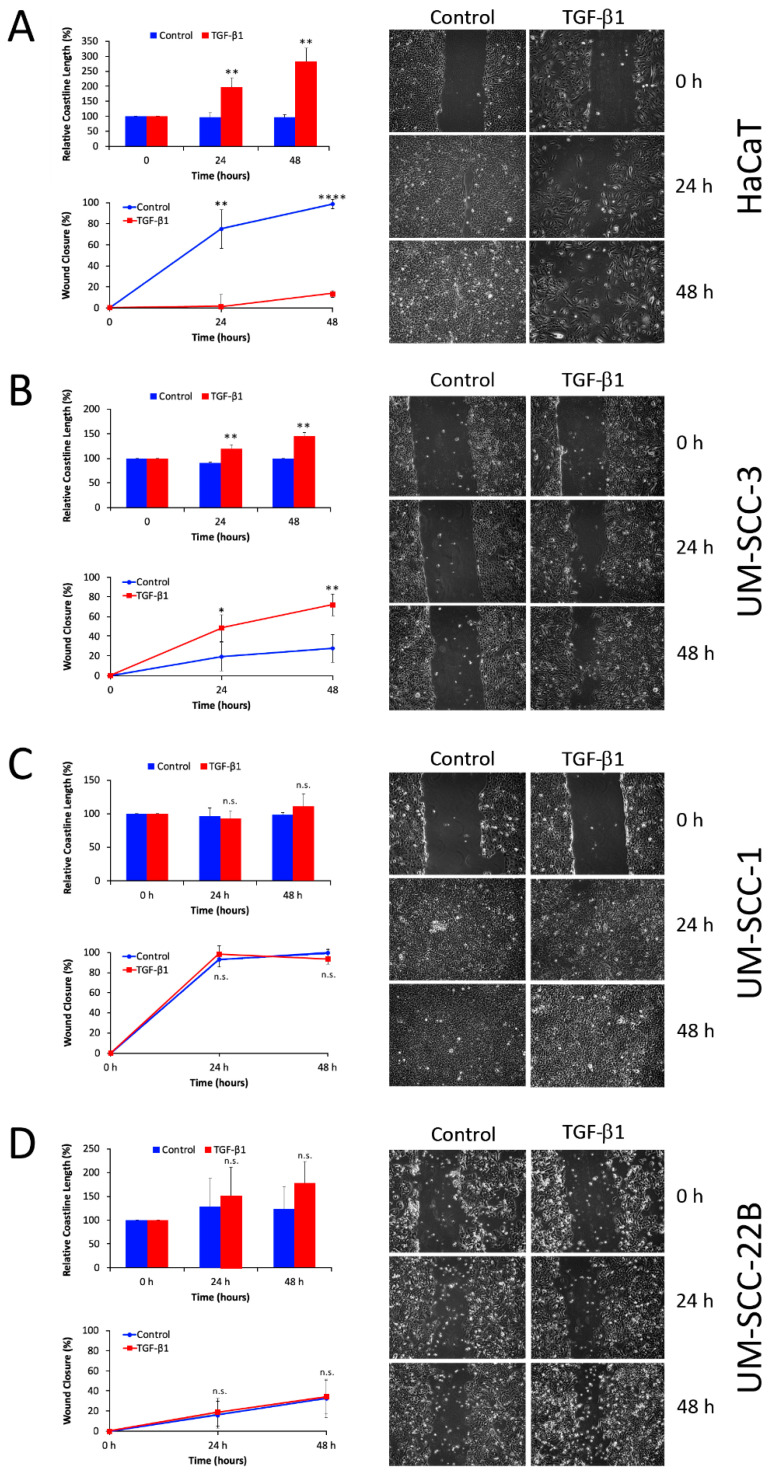
Effect of TGF-β1 on cell motility. Wound closure and relative coastline length were evaluated 24 and 48 hr after TGF-β1 treatment in HaCaT (**A**), UM-SCC-3 (**B**), UM-SCC-1 (**C**), and UM-SCC-22B (**D**) cell lines. Data represent the mean ± SD (*n* = 3), with *p* < 0.05 considered statistically significant. Statistical differences were indicated as *: *p* < 0.05, **: *p* < 0.01, ****: *p* < 0.0001, n.s.: not significant.

A wound closure (scratch) assay was performed to assess the effect of TGF-β1 treatment on the migratory ability of the cell lines (Figure 3). Here, two different features were evaluated: the level of wound closure and the so-called relative coastline length. Significant changes in these parameters were observed in HaCaT and UM-SCC-3 cells, whereas UM-SCC-1 and UM-SCC-22B cells did not exhibit significant differences between TGF-β1 stimulated and unstimulated cell lines. Interestingly, in HaCaT cells, the level of wound closure was significantly diminished after TGF-β1 treatment, which microscopically correlated with a diffuse migratory mesenchymal phenotype (Figure 3A).

**Figure 4:**
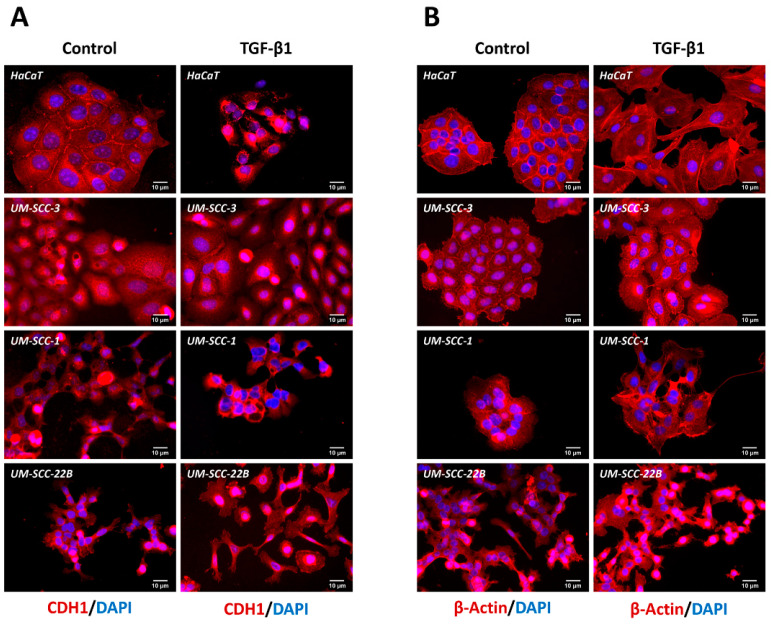
The effect of TGF-β1 on the cellular localization of CDH1 and β-actin. Confocal microscopy depicting CDH1 (**A**) and β-actin (**B**) expression (both red) in HaCaT, UM-SCC-3, UM-SCC-1, and UM-SCC-22B cell lines in the presence or absence of TGF-β1. DAPI (blue) was used for nuclear counterstaining. (No specific signal is seen in cells treated with anti-mouse IgG, see Supplementary Figure S4).

Immunocytochemistry demonstrated CDH1 expression in all HNSCC cell lines. However, HaCaT cells exhibited a pronounced CDH1 cell surface localization, as typically seen in cells with a strong epithelial phenotype. In sharp contrast, UM-SCC-1 and UM-SCC-22B cells did not show this phenotype. In these cell lines, CDH1 was rather intracellular. Furthermore, the morphology of HaCaT and UM-SCC-3 cells appeared more epithelial, compared with UM-SCC-1 and UM-SCC-22B cells (Figure 4A, left). Interestingly, after TGF-β1 exposure, HaCaT cells exhibited the most distinct response, losing their CDH1 surface expression and epithelial phenotype (Figure 4A, right). This effect was less obvious in UM-SCC-1 and UM-SCC-22B cells. Even without exogenous TGF-β1 treatment, they exhibit mesenchymal features. In addition, TGF-β1 treatment morphologically correlated with β-actin reorganization in the tested cell lines, in particular HaCaT (Figure 4B), which was associated with β-actin upregulation (Figure 2).

Next, we performed targeted sequencing in the four tested cell lines to evaluate the mutation status of 50 tumor-related genes (Supplementary Table S1). All four cell lines exhibited *TP53* mutations with predicted loss of function. In addition, UM-SCC-3 cells exhibited *CDKN2A* mutations in both alleles (loss of heterozygosity) (LOH). Interestingly, UM-SCC-22B was the only cell line carrying a *SMAD4* mutation, which was present in both alleles (LOH). Since *SMAD4* mutations are associated with a mesenchymal phenotype, invasion, and metastasis [19] and the UM-SCC-22B cell line exhibited such a phenotype, it was obvious to assume that the *SMAD4* mutation observed in UM-SCC-22B is functionally related to the phenotype of this HNSCC cell line.

To test this assumption, SMAD4 expression constructs were generated by RT-PCR-cloning using RNA derived from UM-SCC-22B and HaCaT control cells. Surprisingly, when sequencing the constructs derived from single bacterial clones, we noticed the presence of a *SMAD4* cDNA species in UM-SCC-22B cells that is carrying an additional 14 bp insertion mutation (Supplementary Figure S5A), resulting in a shift of the open reading frame which is predicting a truncated SMAD4 protein (Supplementary Figure S5B).

**Figure 5:**
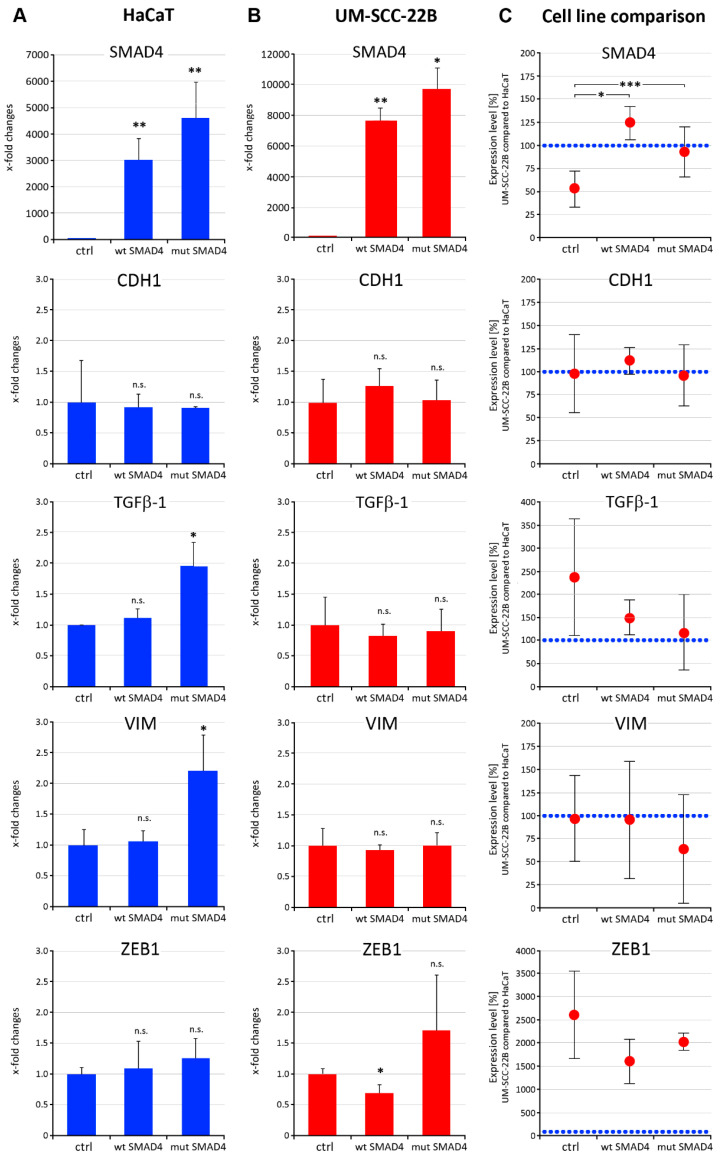

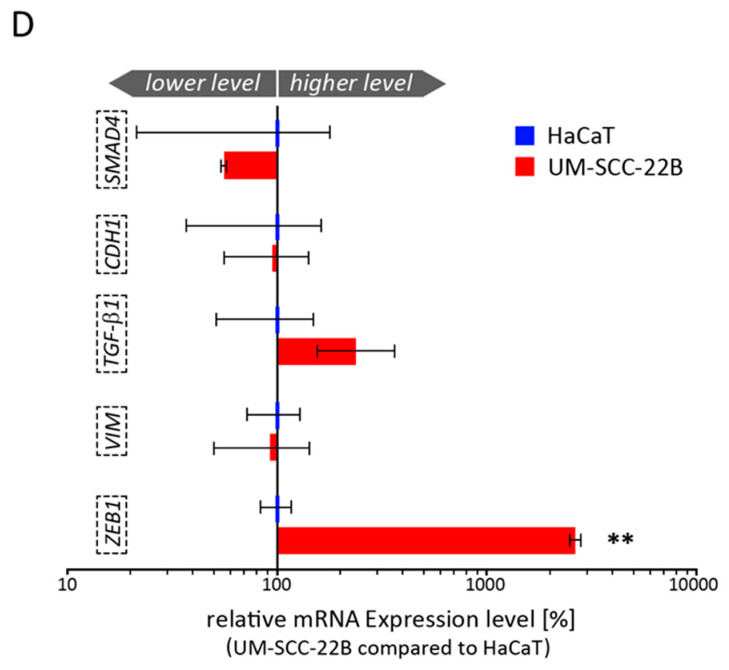
Influence of *SMAD4wt* and *SMAD4mut* overexpression on EMT-related genes. *SMAD4wt* and *SMAD4mut* expressing plasmids were transfected in HaCaT (**A**) and UM-SCC-22B (**B**) cell lines. Gene expression changes were compared against control values (**A**,**B**). (**C**) Gene expression ratios of UM-SCC-22B compared with HaCaT were evaluated in control cells and after transfection with *SMAD4*-expressing plasmids. (**D**) Comparison of basal mRNA expression levels of *SMAD4*, *CDH1*, *TGF-β1*, *VIM,* and *ZEB1* between HaCaT and UM-SCC-22B cells (data correspond to the controls (ctrl) as shown in **A**–**C**). Data represent the mean ± SD (*n* = 3–4), with *p* < 0.05 considered statistically significant. Statistical differences were indicated as *: *p* < 0.05, **: *p* < 0.01, ***: *p* < 0.001, n.s.: not significant.

To evaluate possible functional consequences due to the expression of the truncated SMAD4 protein, we transfected the most epithelial (control) cell line HaCaT and UM-SCC-22B cells with SMAD4wt or SMAD4mut expressing constructs and evaluated changes in the expression levels of relevant genes (*SMAD4*, *CDH1*, *ZEB1*, *TGF-β1*, and *VIM*). High levels of *SMAD4* overexpression could be achieved in both cell lines after transfection of SMAD4wt or SMAD4mut constructs. A significant upregulation of *TGF-β1* and *VIM* transcripts was observed in HaCaT cells transfected with the SMAD4mut construct (Figure 5A). On the other hand, expression of SMAD4wt in UM-SCC-22B cells resulted in a significant reduction in *ZEB1* expression (Figure 5B).

## 4. Discussion

Clinically, tumors with a rather mesenchymal phenotype are associated with a more aggressive course of disease. HNSCC tumors, the most frequent malignancies of the upper aero-digestive tract, are morphologically heterogeneous [31], exhibiting variable tendencies to invade and metastasize. EMT plays a central role in processes such as embryogenesis and wound healing [32] but is also considered paramount for tumor cell invasion and metastasis [33].

**Figure 6:**
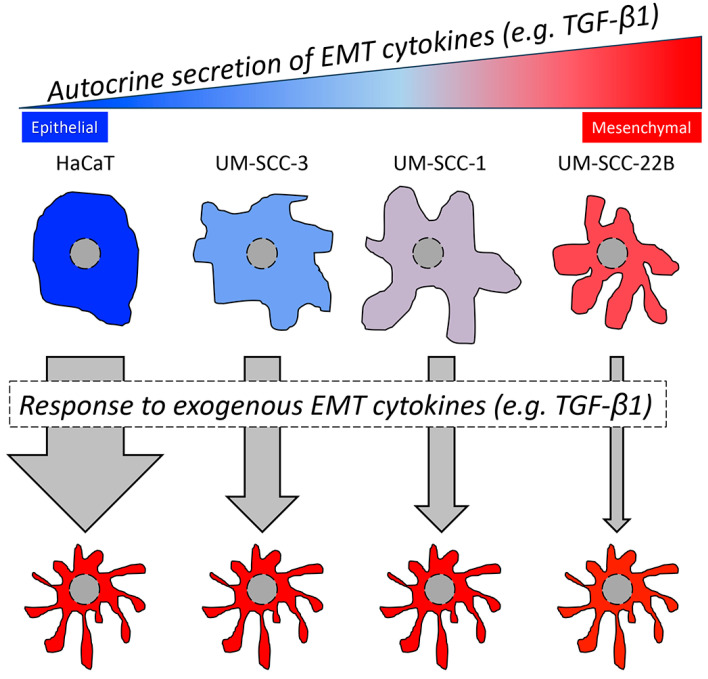
The epithelial or mesenchymal phenotype of HNSCC and HaCaT cells depends on the supply of autocrine and exogenous EMT cytokines.

A major drawback of studying tumor cell lines, in general, is the lack of the TME and TME-associated cells, such as the immune cells and fibroblasts that could influence tumor growth, invasion, and metastasis due to secretion of, e.g., EMT-relevant factors. However, looking directly at the tumor cells, represented by the respective cell lines, also has a major advantage since all experimental observations are directly related to the tumor cell itself that was disposed of the influence from the TME. When evaluating 3 HNSCC cell lines together with a control keratinocyte cell line, we observed dramatic differences in their growth pattern and biological behavior. These differences were related to a more epithelial or mesenchymal phenotype of these cell lines. When comparing the four tested cell lines regarding their epithelial or mesenchymal phenotype, we could order the cell lines as follows (epithelial > mesenchymal): HaCaT > UM-SCC-3 > UM-SCC-1 > UM-SCC-22B (Figure 6). HaCaT is frequently used as a control cell line for studies on HNSCC since it has a pronounced epithelial phenotype and is considered as low or non-invasive [24,34]. It exhibited the highest response to TGF-β1, which is in accordance with previous observations [35,36]. 

Interestingly, UM-SCC-22B, the cell line with the most mesenchymal phenotype, also exhibited a mutation in the *SMAD4* gene. SMAD4, also known as DPC4 (deleted in pancreatic cancer 4), is a crucial and central component of the TGF-β signaling pathway [9]. Wildtype SMAD4 acts as a tumor suppressor [20], but *SMAD4* mutations are commonly found in tumors and are associated with tumor progression and metastatic spread. Our observations of SMAD4 mutations in UM-SCC-22B cells are in line with these reports since, compared to HaCaT cells, UM-SCC-22B exhibited significantly higher mRNA levels of the mesenchymal transcription factor ZEB1 [37] and, on average, higher TGF-β1 transcript levels. It appears that UM-SCC-22B cells are already activated, i.e., partly transitioned to a mesenchymal phenotype. Furthermore, transfection of wt *SMAD4* could significantly reduce ZEB1 levels in UM-SCC-22B, whereas transfection of HaCaT cells with mutant SMAD4 significantly induced TGF-β1 and vimentin levels.

## 5. Conclusions

Supposedly low invasive HNSCC cells, under certain circumstances, can likely switch to a higher invasive phenotype and vice versa. It is conceivable that changes in the TME, such as induction or reduction of inflammatory cells and of cancer-associated fibroblasts (CAFs), which are known to secrete EMT-relevant cytokines, could have a major impact on tumor progression and metastatic spread [38]. These observations are in accordance with the concept of pro- and anti-tumorigenic inflammatory conditions [39].

## Data Availability

The original contributions presented in the study are included in the article/Supplementary Material; further inquiries can be directed to the corresponding author.

## Supplementary Materials

The following supporting information can be downloaded at https://www.mdpi.com/article/10.3390/cancers16183172/s1, Figure S1: Real-time cell analysis; Figure S2: Effect of tested cytokines on CDH1 downregulation in HaCaT, UM-SCC-3, UM-SCC-1 and UM-SCC-22B cell lines; Figure S3: Uncropped Western blots; Figure S4: No specific signal is seen in cells treated with anti-mouse IgG; Figure S5: Sequence analysis of SMAD4; Table S1: Evaluation of gene mutations in the tested cell lines using the Cancer Hotspot Panel v2.
