# Supplementary material for: The Role of TGF-β1 and Mutant SMAD4 on Epithelial-Mesenchymal Transition Features in Head and Neck Squamous Cell Carcinoma Cell Lines
Source: Cancers (Basel). 2024 Sep 16;16(18):3172. doi: 10.3390/cancers16183172 (PMC11429651; doi:10.3390/cancers16183172)
Supplement: Supplementary file 1 [file cancers-16-03172-s001.zip › cancers-3164746-supplementary.pdf]

Supplementary Materials

The Role of TGF-β1 and Mutant SMAD4 on Epithelial-Mesenchymal Transition Features in Head and Neck Squamous Cell Carcinoma Cell Lines

Michael Bette [1,†] , Laura Reinhardt [2,†], Uyanga Gansukh [2], Li Xiang-Tischhauser [2] , Haifa Meskeh [2], Pietro Di Fazio [3], Malte Buchholz [4], Boris A. Stuck [2] and Robert Mandic [2,*]

1 Institute of Anatomy and Cell Biology, Philipps-Universität Marburg, 35037 Marburg, Germany
2 Department of Otorhinolaryngology, Head and Neck Surgery, University Hospital Marburg, Philipps-Universität Marburg, 35043 Marburg, Germany
3 Department of Nuclear Medicine, Philipps-Universität Marburg, 35043 Marburg, Germany
4 Clinic for Gastroenterology, Endocrinology and Metabolism, University Hospital, Philipps-Universität Marburg, 35043 Marburg, Germany
* Correspondence: mandic@med.uni-marburg.de; Tel.: +49-6421-5861400; Fax: +49-6421-5862421
† These authors contributed equally to this work.

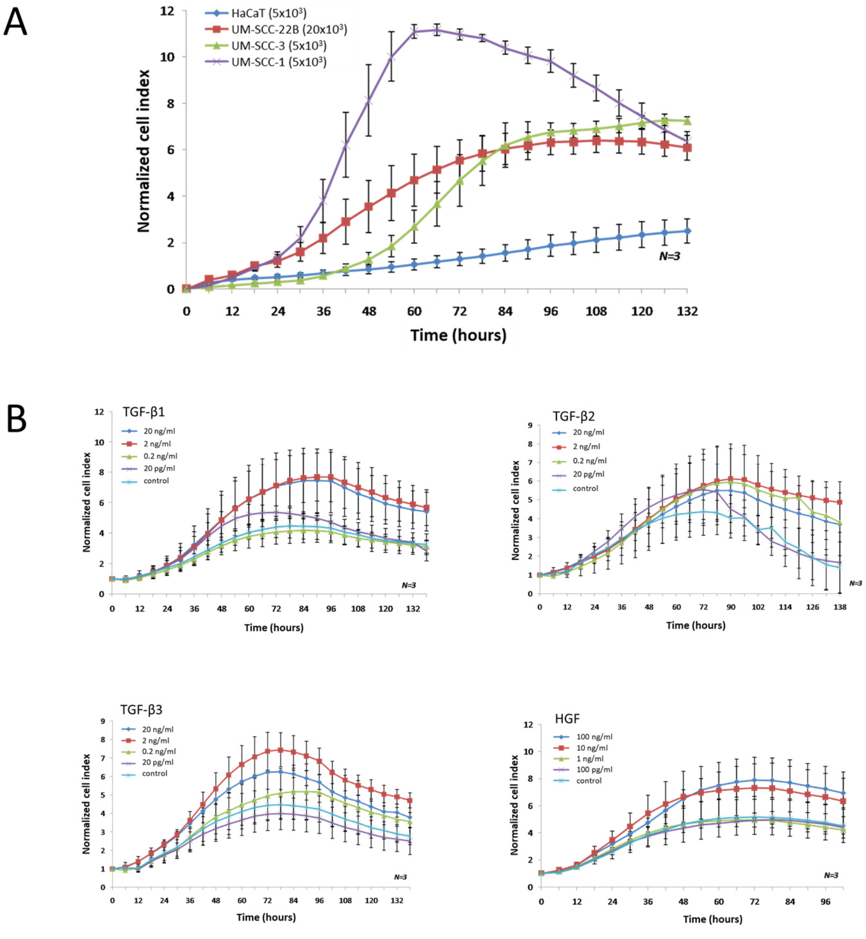

Figure S1. Real-time cell analysis. (A) Comparison of cell growth levels (normalized cell index) between HaCaT, UM-SCC-3, UM-SCC-1, and UM-SCC-22B cell lines. (B) Measuring cell growth responsiveness to different concentrations of TGF-β1, -β2, -β3, and HGF in UM-SCC-3 cells.

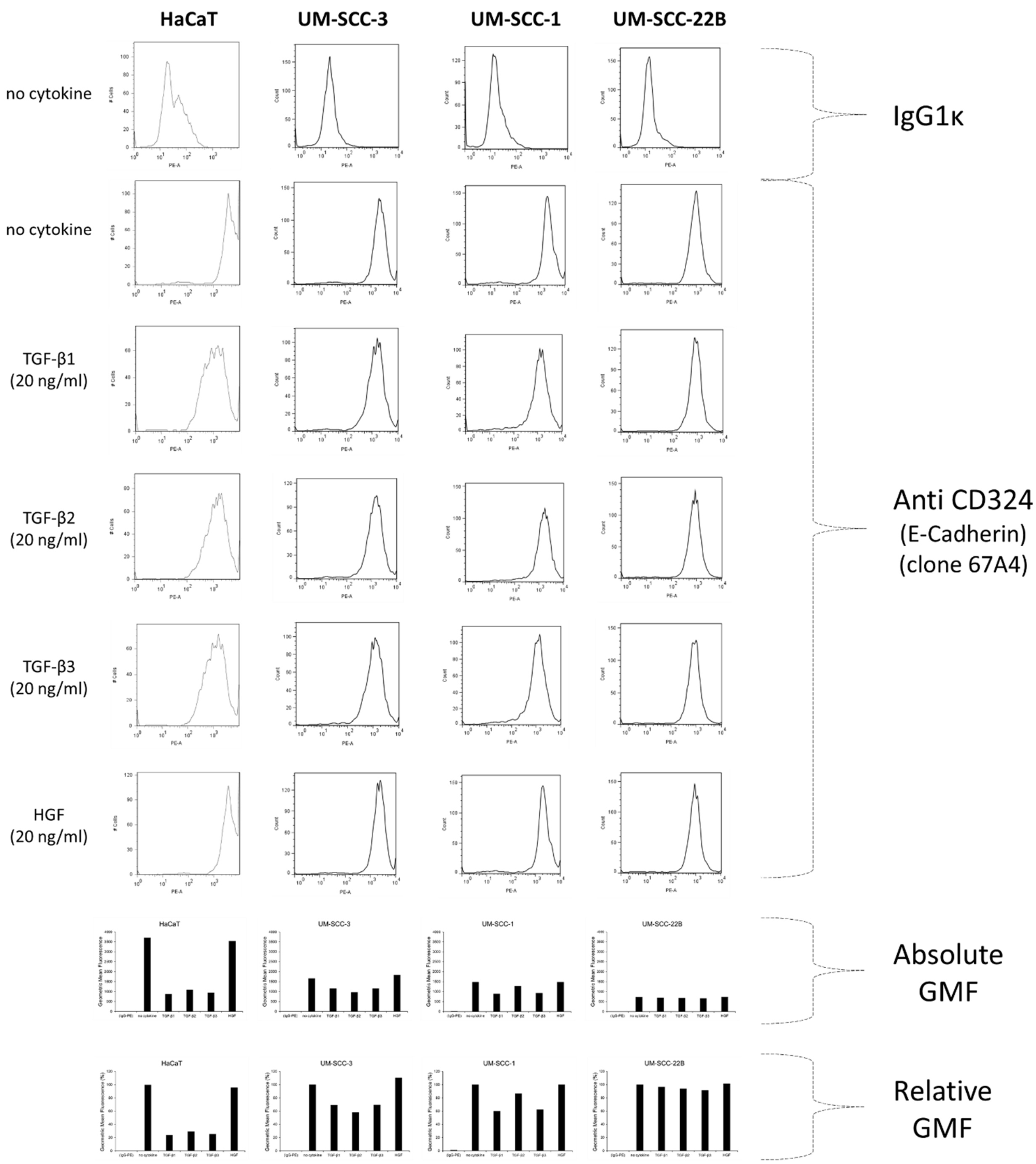

Figure S2. Effect of tested cytokines on CDH1 downregulation in HaCaT, UM-SCC-3, UM-SCC-1, and UM-SCC-22B cell lines. When monitoring CDH1 surface expression after treatment with 20 ng/ml TGF-β1, -β2, -β3, or HGF, the most pronounced CDH1 surface downregulation (CDH1-geometric mean fluorescence, CDH1-GMF) was observed in HaCaT cells after exposure to TGF-β1,-β2, -β3, and less so in UM-SCC-3 and UM-SCC-1 cells, whereas the UM-SCC-22B cell line did not exhibit any notable response. None of the tested cell lines responded to treatment with HGF. (Exploratory investigation, n=1).

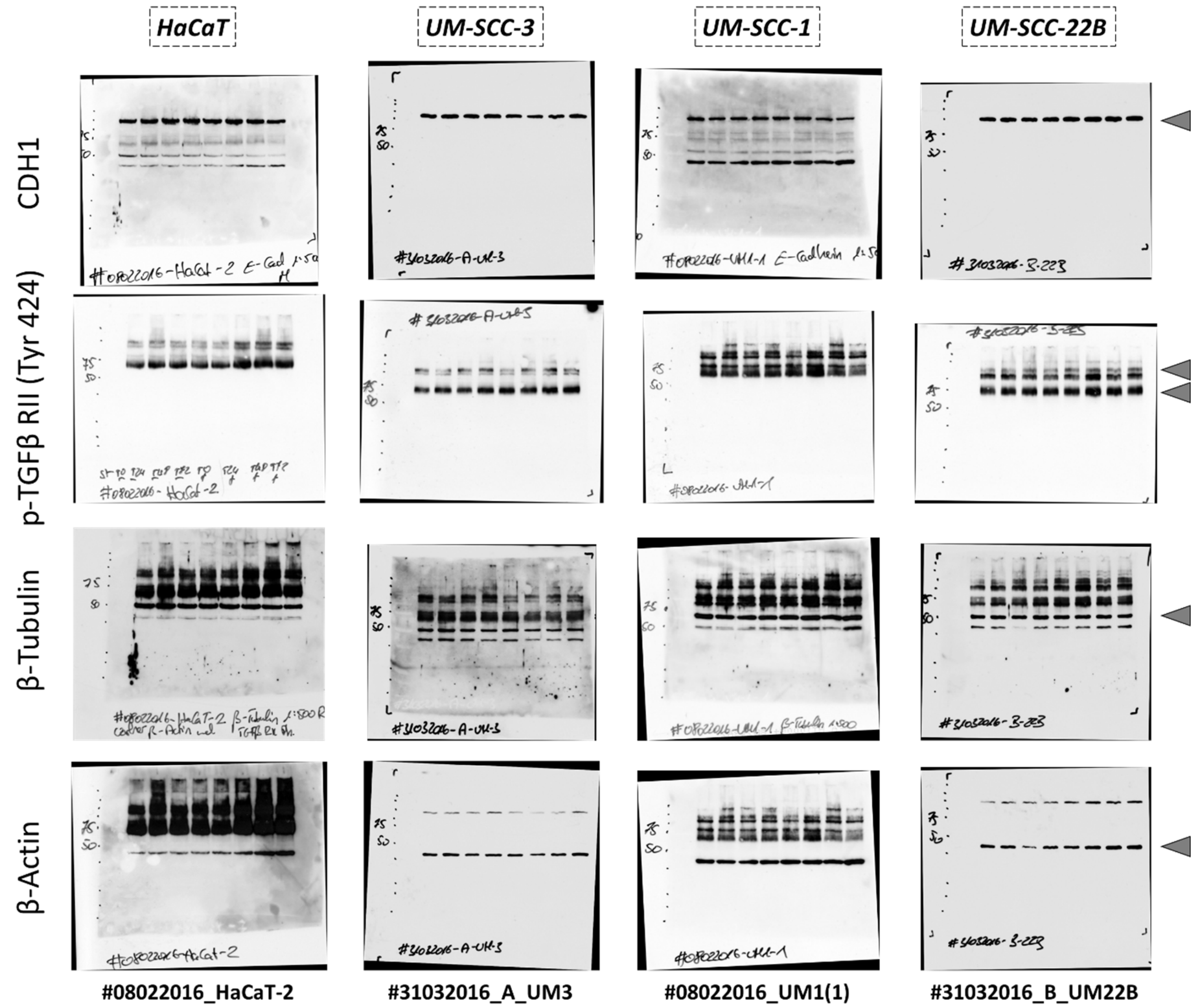

Figure S3. Uncropped Western blots. Shown are the uncropped Western blot images as depicted in Figure 2. Arrows point to the position of the bands corresponding to the tested protein of interest.

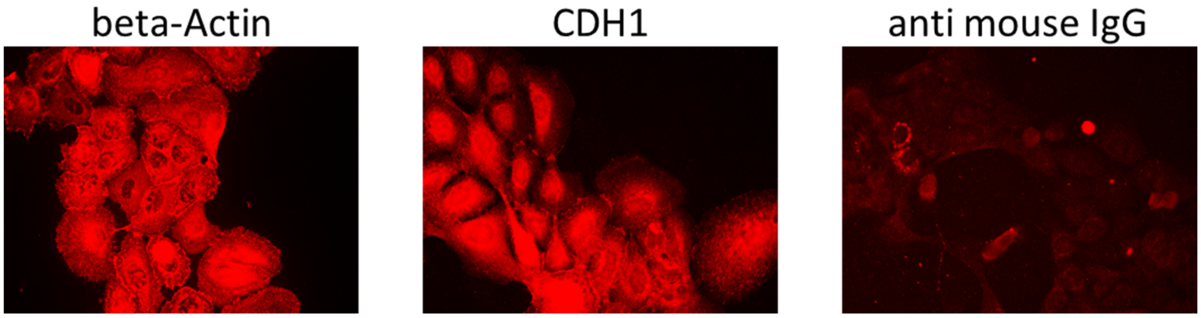

Figure S4. Control staining to Figure 4. No specific signal is seen in cells treated with anti-mouse IgG (image of UM-SCC-3 cells treated with TGF-β1)

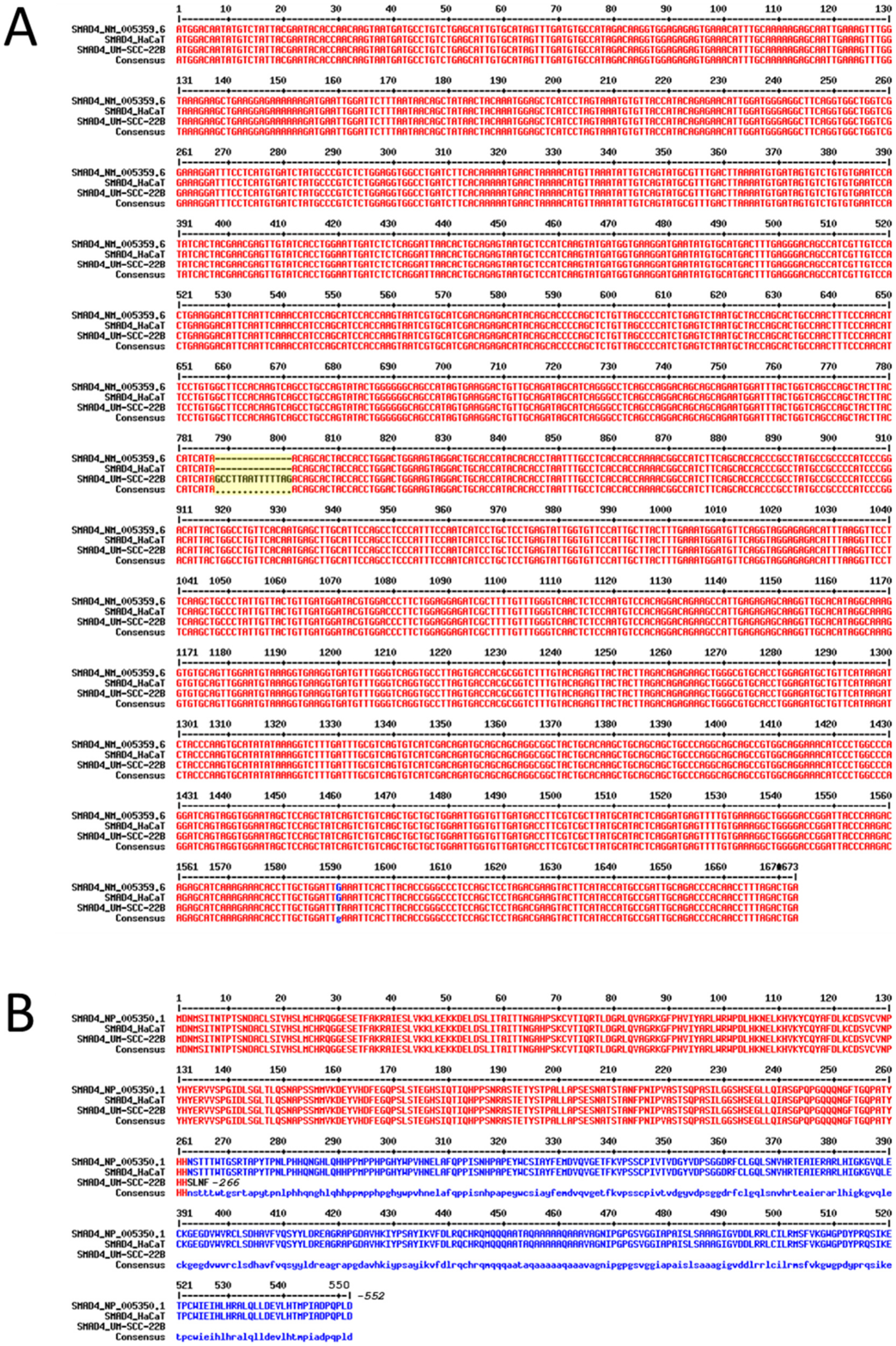

Figure S5. Sequence analysis of SMAD4. (A) Nucleotide sequence of a clone derived from UM-SCC-22B cells showing an additional 14 bp frameshift insertion next to the c.1576G>T mutation as observed during targeted sequencing using the Cancer Hotspot Panel v2 as listed in Table S1 (note that the numbering in "A" is 1590 due to the additional 14 bp insertion). (B) The frameshift mutation gives rise to a truncated SMAD4 protein of 266 aa (wt=552 aa) carrying 4 new aa (-SLNF) at its C-terminal end. Sequence alignment was performed with the online tool "MultAlin" (http://multalin.toulouse.inra.fr/multalin/; Corpet F., Nucl Acids Res, 1988, 10881-10890).

Table S1. Evaluation of gene mutations in the tested cell lines using the Cancer Hotspot Panel v2.

Gene	coding	cell line<br>alle freq.(%)	variant	impact	protein effect	pathogenic	SNP	Description
EGFR	c.2361G>A	UM-SCC-1 (52.18)<br>UM-SCC-22B (99.90)<br>UM-SCC-3 (39.82)<br>HaCaT (55.08)	Q787=	-	-	**yes**	yes	
CDKN2A	c.262G>T	UM-SCC-3 (99.50)	E88*	nonsense	loss of function	**yes**	no	
FGFR3	c.1953G>A	UM-SCC-1 (99.80)<br>UM-SCC-22B (99.68)<br>UM-SCC-3 (100.00)<br>HaCaT (100.00)	T651=	-	-	**yes**	yes	
RET	c.2307G>T	UM-SCC-1 (51.90)<br>UM-SCC-22B (100.00)<br>UM-SCC-3 (100.00)<br>HaCaT (100.00)	L769=	-	-	**yes**	yes	
SMAD4	c.1576G>T	UM-SCC-22B (100.00)	E526*	nonsense	truncated	**yes**	no	
TP53	c.559+1G>A	UM-SCC-1 (100.00)			truncated	**yes**	no	Affecting splicing
TP53	c.659A>G	UM-SCC-22B (49.85)	Y220C	missense	loss of function	**yes**	no	Hotspot mutation in DNA binding domain
TP53	c.743G>A	UM-SCC-3 (99.90)	R248Q	missense	loss of function	**yes**	no	Hotspot mutation in DNA binding domain
TP53	c.535C>T	HaCaT (67.72)	H179Y	missense	loss of function	**yes**	no	within DNAbinding domain
TP53	c.843CC>TT	HaCaT (49.88)	D281=<br>R282W	-<br>missense	-<br>loss of function	no<br>yes	no<br>no	Hotspot mutation in DNA binding domain
NOTCH1	c.5035G>T	UM-SCC-22B (100.00)	E1679*	nonsense	truncated	maybe	no	
KIT	c.1621A>C	UM-SCC-22B (99.94)<br>UM-SCC-3 (33.87)	M541L	missense	unknown	unknown	yes	transmembrane domain
PIK3CA	c.352+40A>G	HaCaT (49.36)				unknown	no	
	c.1173A>G	HaCaT (51.26)	I391M	missense		unknown	yes	
	c.2712C>G	UM-SCC-22B (48.37)<br>UM-SCC-3 (54.68)	S904=	-	-		yes	
SMARCB1	c.727C>T &<br>c.1119-41G>A	UM-SCC-22B (41.07)	Q243*	nonsense		unknown	no<br>no	Common intron variant
TP53	c.215C>G	UM-SCC-22B (99.11)<br>UM-SCC-3 (97.75)<br>HaCaT (96.83)	P72R	missense	unknown	unknown	yes	
FLT3	c.1310-3T>C	UM-SCC-1 (99.95)<br>UM-SCC-22B (67.87)<br>UM-SCC-3 (100.00)<br>HaCaT (51.12)				no	no	Known intronic varants
KDR	c.798+54G>A	UM-SCC-1 (49.02)<br>UM-SCC-22B (52.56)<br>UM-SCC-3 (37.35)<br>HaCaT (100.00)					no	Known intronic varants, splice site
	c.1416A>T	UM-SCC-1 (48.22)<br>UM-SCC-3 (99.95)	Q472H			no	yes	
	c.2615-36A>CA	UM-SCC-1 (100.00)<br>UM-SCC-3 (42.72)<br>HaCaT (100.00)						Known intronic varants
PDGFRRA	c.1701A>G	UM-SCC-1 (99.90)<br>UM-SCC-22B (99.93)<br>UM-SCC-3 (99.81)<br>HaCaT (100.00)	P567=	-	-		yes	
STK11	c.465-51T>C	UM-SCC-1 (99.89)					yes	Common intron variant

No sequence variations were observed in the following genes *ABL1, AKT1, ALK, ATM,BRAF, CDH1, CTNNB1, ERBB2, EZH2, FBXW7, FGFR1, FGFR2, GNA11, GNAQ, GNAS, HNF1A, IDH1, IDH2, JAK2, JAK3, KRAS, MET, MLH1, MPL, NPM1, NRAS, PTEN, PTPN11, RB1, SMO, SRC, VHK*
